# Changes of lysosome by L-serine in rotenone-treated hippocampal neurons

**DOI:** 10.1186/s42649-022-00084-z

**Published:** 2023-01-10

**Authors:** Sumin Shin, Su-Kyeong Hwang, Ji Young Mun

**Affiliations:** 1 Neural Circuit Research Group, Korea Brain Research Institute, Daegu, South Korea; 2 Department of Pediatrics, School of Medicine, Kyungpook National University, Daegu, 41944 South Korea; 3 Astrogen Inc., 440, Hyeoksin-daero, Dong-gu, Daegu, 41072 South Korea

**Keywords:** L-serine, Reactive oxygen species, Mitochondria, Lysosome

## Abstract

Oxidative stress destroys cellular organelles and damages DNA, eventually leading to degenerative brain disorders. Persistent mitochondrial damage by oxidative stress eventually causes cells to inhibit the function of lysosomes. Rotenone used in this study inhibits complex 1 of the mitochondrial electron transport chain. Due to this inhibition, the production of free radicals is promoted, and oxidative stress can occur. To test as a role of antioxidant, L-serine was treated before treatment of rotenone to HT22 hippocampal cells. Then, changes in the activity and structure of lysosomes were analyzed. As a result, the oxidative stress caused by rotenone in HT22 cells was protected by L-serine. L-serine reduced free radicals in cells, and the damaged lysosomal structure and lysosome activity were also protected.

## Introduction

Oxidative stress is considered as one of the major factors in the induction of neurodegenerative diseases, which are associated with an imbalance between antioxidants and reactive oxygen species (ROS). Rotenone was used to induce oxidative stress in mitochondria, because it inhibits complex I of the mitochondrial electron transport chain (ETC). This promotes the production of ROS, which can eventually lead to degenerative brain disease. Hence, rotenone has been widely used as a chemical that induces oxidative stress (Sanders and Greenamyre 2013). The generation of ROS is commonly proposed as the constant origin of cellular oxidative stress, and also as a significant factor in the pathophysiological processes, specifically aging and its related neurodegenerative diseases (Panee, Liu et al. 2007). The major ROS-generating site in HT22 cells, a mouse hippocampal neuron, is complex I of the mitochondrial ETC. This cell line is a representative model for studying the interactions of inflammation and oxidative stress in the central nervous system (Panee, Liu et al. 2007, Günaydin, Çelik et al. 2021).

Serine, an amino acid, exists in two forms, D-serine and L-serine. L-serine can be synthesized in the body and can also be obtained in the diet. D-serine is synthesized from L-serine in neurons. By playing a significant role as a protein kinase-mediated phosphorylation site on proteins, L-serine functionally manages diverse proteins. Additionally, in various metabolic pathways, L-serine is a precursor to produce some necessary cellular molecules that are required for the synthesis of neurotransmitters, phospholipids, and nucleic acids (Snell and Persoone 1989). L-serine is also required for cell proliferation, and the synthesis of L-serine and its by-products plays an important role in the differentiation and survival of neurons. Furthermore, for the reason that downstream metabolites of L-serine, such as glycine and cysteine are precursor amino acids required for the synthesis of the antioxidant glutathione, which protects cells from oxidative damage (Zhou, He et al. 2017). It is a safe substance approved by the FDA, and it has been used to treat epilepsy and psychiatric disorders. The therapeutic effects and safety of neurodegenerative diseases have been reported through animal experiments and clinical trials (Furuya 2008, Ren, Qiang et al. 2013, Sun, Qiang et al. 2014, Lisha Ye 2021). The protective effect of L-serine on mitochondrial damage and apoptosis caused by DMNQ-induced ROS in HT22 cells, a mouse hippocampal neuron, has been reported (Kim, Hwang et al. 2019). However, the mechanism study of the therapeutic effects against damage caused by oxidative stress was not still enough. Therefore, the neuronal protective effects of L-serine from oxidative stress were investigated by observing the structures of cellular organelles after treatment with L-serine in this study. In our previous paper, lysosomal defects caused by propionic acid, were restored by L-serine in HT22 cells and mouse primary hippocampal neurons (Jeon et al. 2022). Autophagic cell death and autophagy can be induced by rotenone through the inhibition of autophagic flux (Janda, Isidoro et al. 2012, Mader, Pivtoraiko et al. 2012) or ROS-mediated mechanism (Chen, McMillan-Ward et al. 2007). In this study, the effects of L-serine on the lysosomal dysfunctions were analyzed in rotenone treated neuron through microscopy techniques.

## Materials and methods

### Cell culture

The murine hippocampal neuronal cell line HT22 was obtained from Korean Cell Line Bank (Seoul, Korea) and maintained in Dulbecco’s Modified Eagle’s Medium containing high glucose and sodium pyruvate (Gibco, Waltham, MA, USA) supplemented with 10% fetal bovine serum (Gibco, Waltham, MA, USA), and 0.1% of penicillin-streptomycin in 5% CO_2_ at 37 °C.

### CCK-8 assay

HT22 cells were seeded at a density of 6 × 10^4^ in 24-well cell culture plates (Corning Inc., USA). Then, incubated at 37 °C in a humidified atmosphere with 5% CO_2_. After 24 h, 10 mM L-serine was treated at 5 h before 1 μM rotenone treatment. 1 μM Rotenone was treated for 19 h. Then, 10 μL of the CCK-8 reagent (Dojindo, Japan) was added into each well, and after incubation for 2 h, cell culture supernatant was divided by 100 μL per well into 96-well cell culture plates (Corning Inc., USA). Optical density (OD) at 450 nm was measured using a VersaMax Microplate Reader (Molecular Devices, USA). The percentage that each concentration accounted for in comparison with the control was presented as cell viability.

### Intracellular ROS assay

The intracellular content of ROS was determined by measuring the fluorescence intensity of 2′, 7′-dichlorofluorescein. Cells were grown in 35 mm glass-bottomed culture dishes (NEST Biotechnology Co., Wuxi, China) to 60%–70% confluency. On the next day, to visualize ROS, H_2_DCFDA (Thermo Fisher Scientific, Waltham, MA, USA) was added to the cell medium at a final concentration of 5 μM and incubated at 37 °C for 20 min. The cells were then washed carefully with a phosphate-buffered solution. Immediately, cells were observed under a confocal light microscope (Ti-RCP, Nikon, Japan). Fluorescence intensity was measured using ImageJ software (https://imagej.net/software/fiji/downloads).

### Mitochondrial ROS assay

HT22 cells were cultured in 35 mm glass-bottomed culture dishes (NEST Biotechnology Co., Wuxi, China) to 50%–60% confluency. The next day, 10 mM L-serine was treated at 5 h before rotenone treatment. In addition, 1 μM rotenone was treated for 19 h. Then, cells were loaded with the mitochondrial superoxide indicator MitoSOX Red (5 μM, 20 min) and 2′,7′-dichlorodihydrofluorescein diacetate (H_2_DCFDA) (5 μM, 20 min), and then imaged under a confocal light microscope (Ti-RCP, Nikon, Japan). Fluorescence intensity was measured using ImageJ software version 1.51j8.

### Transmission electron microscopy (TEM)

HT22 cells were cultured in 35 mm glass-bottomed culture dishes (NEST Biotechnology Co., Wuxi, China) to 50%–60% confluency. The next day, 10 mM L-serine was treated at the 5 h before rotenone treatment. 1 μM Rotenone was treated for 19 hours. Then, cells were fixed in 2.5% glutaraldehyde-mixed 2% paraformaldehyde solution for 1 h followed by post-fixation in 2% osmium tetroxide for 1 h at 4 °C. The block was stained in 2% uranyl acetate O/N. Subsequently, the cells were dehydrated in an ethanol series and then embedded in Poly/Bed 812 resin (EMS, Hatfield, PA, USA). Embedded samples were sectioned (70 nm) with an ultra-microtome (Leica Microsystems, Wetzlar, Germany), and the sections were then viewed on a Tecnai 20 TEM (Thermo Fisher Scientific, Waltham, MA, USA) at 120 kV. They were then double stained with UranyLess (EMS, Hatfield, PA, USA) for 2 min and with 3% lead citrate (EMS, Hatfield, PA, USA) for 1 min. Images were captured with a US1000X-P camera 200. The acquired images were stitched together using Photomontage software (Thermo Fisher Scientific, Waltham, MA, USA).

### Lysosomal intracellular activity assay

The intracellular lysosomal activity was determined by measuring the fluorescence intensity of lysosome-specific self-quenching substrate. Cells were grown in 35 mm glass-bottomed culture dishes (NEST Biotechnology Co., Wuxi, China) to 60–70% confluency. On the next day, to visualize lysosomal activity, 10 μL of a lysosome-specific self-quenching substrate (Abcam, Cambridge, UK) was added to 1 mL of cell medium and incubated at 37 °C for 1 h. The cells were then washed twice carefully with ice-cold 1X assay buffer (Abcam, Cambridge, UK) containing the tested compound at the same concentration. Immediately, cells were observed under a confocal light microscope (Ti-RCP, Nikon, Japan). Fluorescence intensity was measured using ImageJ software.

### Statistical analysis

Unless otherwise indicated, data are representative of at least three independent experiments. All data are expressed as mean value ± standard error of mean. Statistical analyses were performed using one-way ANOVA and Tukey’s multiple-comparisons test to evaluate the significance of differences between four groups.

## Results

### Effects of L-serine on the intracellular ROS of damaged HT22 cells

**Fig. 1:**
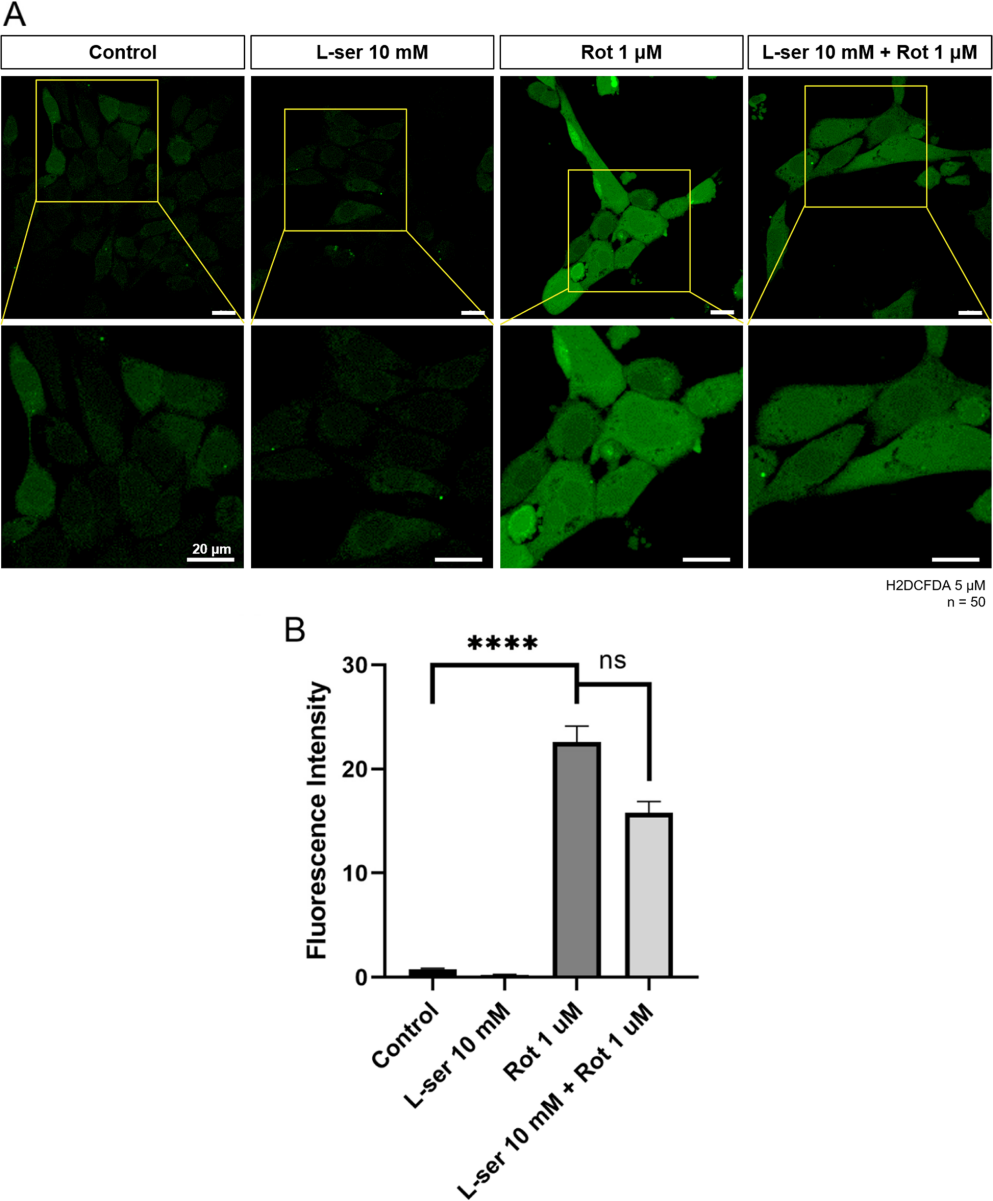
L-serine attenuates cellular ROS production induced by rotenone. HT22 cells were treated with 10 mM of L-serine for 24 h, 1 μM of rotenone for 19 h and pre-treatment10 mM L-serine for 5 h plus 1 μM of rotenone for 19 h, respectively. Cells were then incubated with 5 μM of oxidation-sensitive fluorescent probe H_2_DCFDA for 20 min before observation under confocal laser microscopy. **A** L-serine significantly reduced rotenone-induced intracellular ROS in HT22 cells. The under image panel is enlarged from the upper yellow box. **B** Relative quantification of H_2_DCFDA fluorescence intensity was analyzed using Image J software from 50 cells, respectively. Scale Bar = 20 μm. One-way ANOVA, ****p < 0.0001

To investigate whether L-serine regulates rotenone-induced ROS in HT22 cells and has an antioxidative effects, cellular ROS status were analyzed. H_2_DCFDA assay was used to measure cellular ROS. Compared to the untreated control group, the fluorescence intensity of the group that was treated with only L-serine decreased by half, whereas in the group treated with rotenone, the fluorescence intensity increased about 25 times compared to the control group. This was significantly reduced by about 0.3 times compared to the group treated with rotenone alone, when we treated L-serine before rotenone treatment (Fig. 1A, B).

**Fig. 2:**
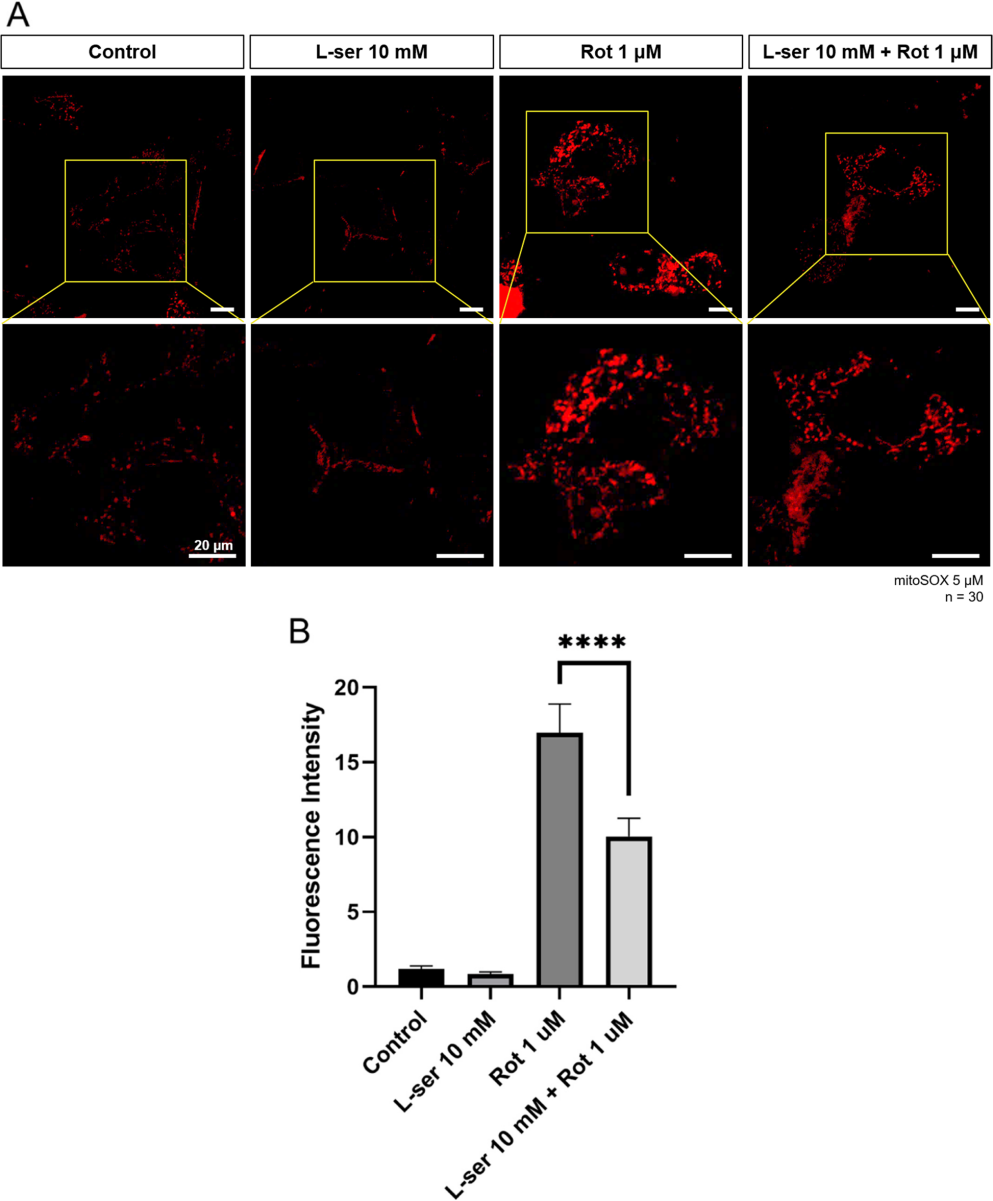
L-serine attenuates mitochondrial ROS induced by rotenone. Mitochondria superoxide was detected with 5 μM of MitoSOX with confocal microscopy in HT22 cells. HT22 cells were treated with 10 mM of L-serine for 24 h, 1 μM of rotenone for 19 h and pre-treatment10 mM L-serine for 5 h plus 1 μM of rotenone for 19 h, respectively. **A** Confocal fluorescence images were taken at 20 min after the addition of MitoSOX. The ROS level was significantly increased by rotenone treatment, But decreased by L-serine treatment. The under image panel is enlarged from the upper yellow box. **B** Relative quantification of MitoSOX red fluorescence measured by Image J software from 30 cells, respectively. Scale Bar = 20 μm. One-way ANOVA, ****p < 0.0001

Then, mitochondrial ROS was analyzed in each group. The L-serine alone treatment group decreased about 0.1 times compared to the control group, and the rotenone alone treatment group increased by 17 times compared to the control group. This was significantly reduced by about 0.4 times compared to the group treated with rotenone alone, when we treated L-serine before treatment of rotenone (Fig. 2A, B).

### The addition of L-serine affects the lysosomal structure and activity of damaged HT22 cells

**Fig. 3:**
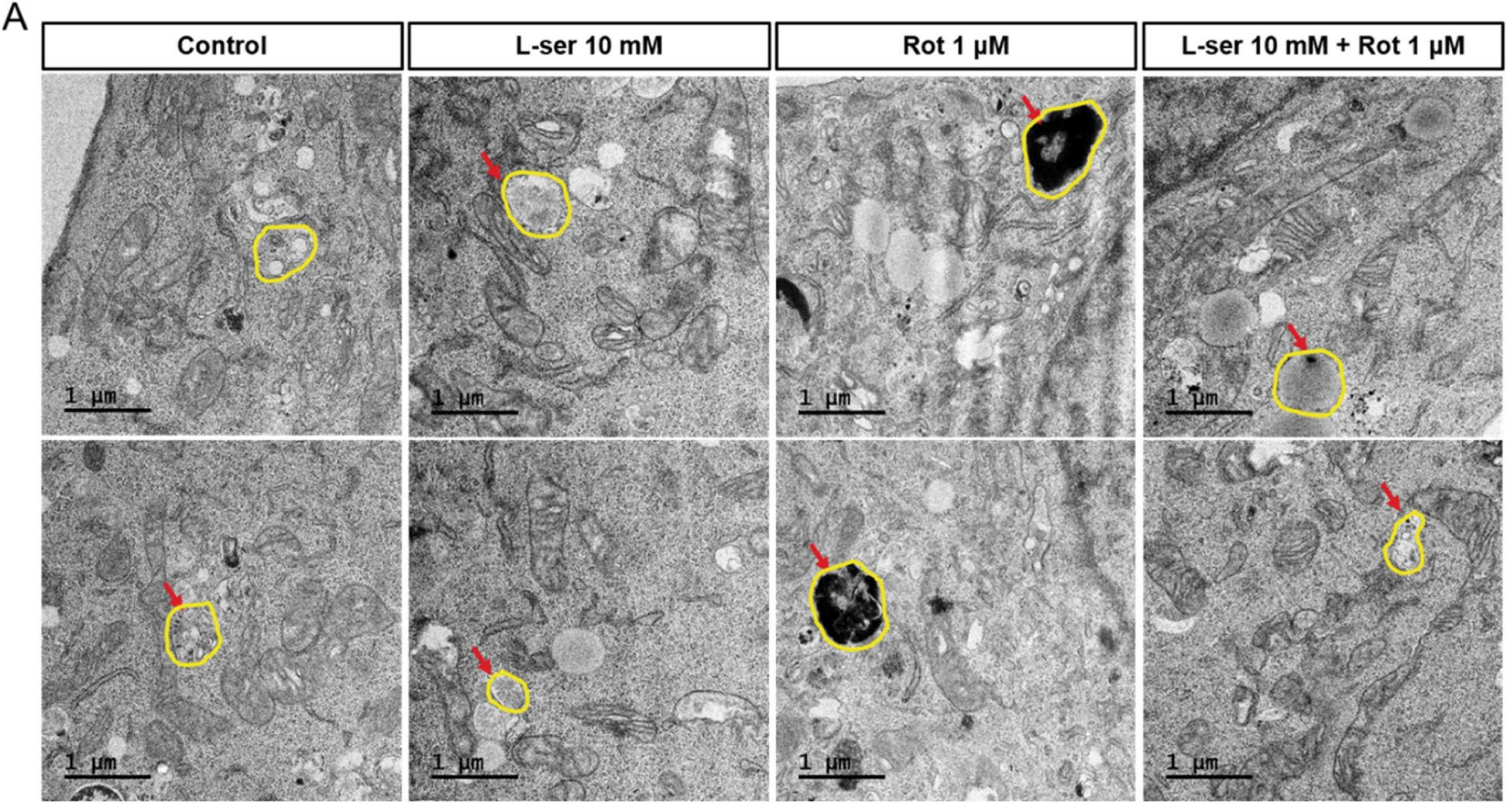
L-serine changes the ultrastructural morphology of lysosomes in rotenone-induced HT22 cell damage. TEM analysis shows that the change of the ultrastructural lysosome morphology after rotenone treatment. Representative TEM micrographs showing the ultrastructure of healthy lysosomes (control, L-serine treated group and pre-treatment L-serine plus rotenone treated group) and high-electron dense lysosomes (rotenone treated group)

**Fig. 4:**
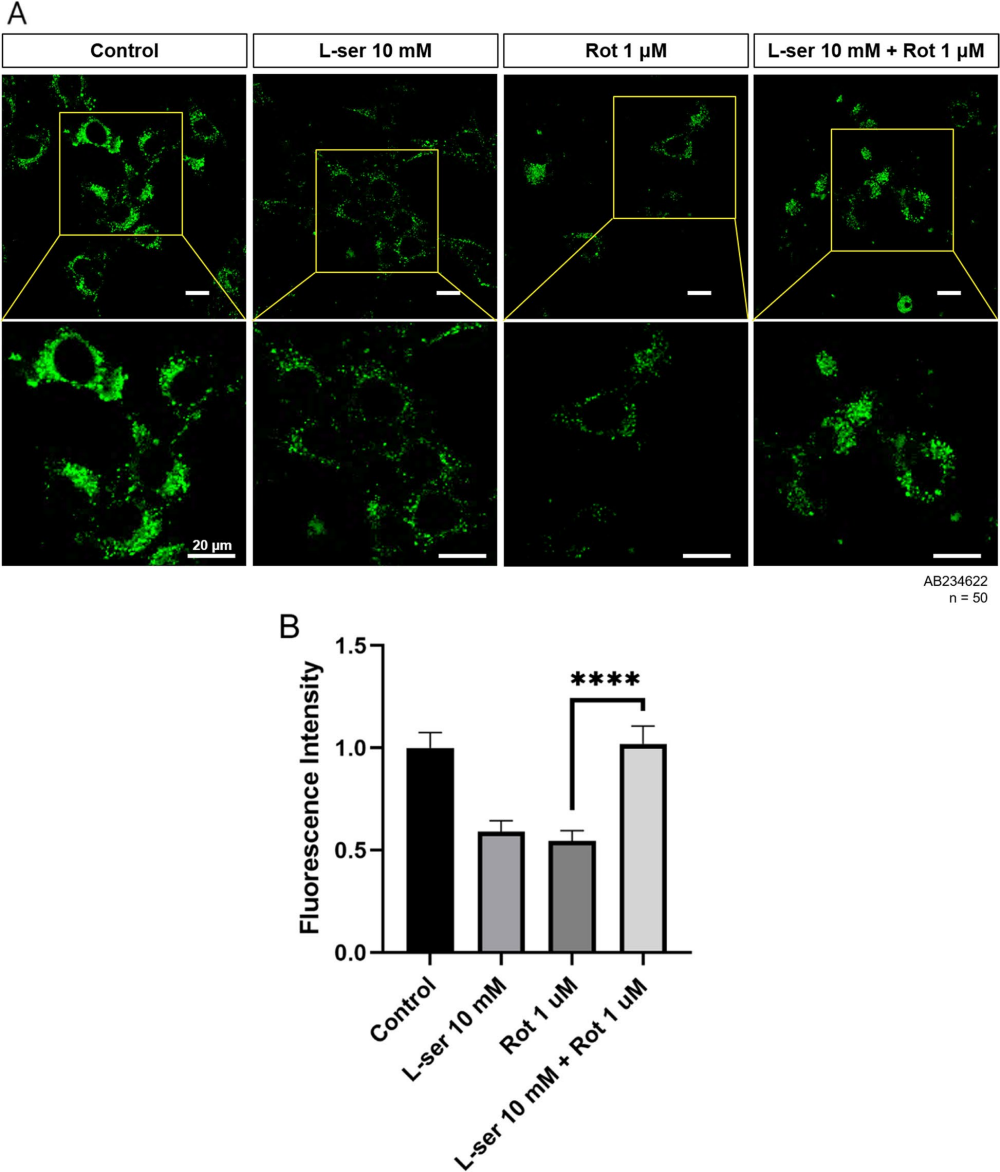
L-serine promotes lysosomal intracellular activity in rotenone-induced damaged HT22 cells. HT22 cells were treated with 10 mM of L-serine for 24 h, 1 μM of rotenone for 19 h and pre-treatment10 mM L-serine for 5 h plus 1 μM of rotenone for 19 h, respectively. Cells were then incubated with lysosomal activity assay kit followed by manufacturer’s manual before observation under confocal laser microscopy. (**B**) Relative quantification of self-quenched substrate fluorescence measured by Image J software from 50 cells, respectively. Lysosomal activity was significantly decreased in the rotenone-treated group, and the decrease was recovered as much as the control in the L-serine pre-treatment group. Scale Bar = 20 μm. One-way ANOVA, ****p < 0.0001

Lysosome activity is important for autophagic process. The TEM analysis of ultrastructural lysosomal morphology showed the changes between autolysosomes after rotenone and L-serine treatment. Untreated control group, 1 μM of rotenone treated group, and 10 mM of L-serine pre-treated groups showed differences in the electron dense of autolysosome. Compared to the group treated with rotenone alone, the lysosomes in the L-serine pretreatment group showed that the substrates were degraded through electron density (Fig. 3A). It was correlated with lysosomal activity, which was investigated to confirm the lysosomal defect observed by electron microscopy. Lysosomal activity was significantly decreased in the rotenone-treated group compared to the control group, and the decrease was recovered as much as the control in the L-serine pretreatment group (Fig. 4A, B).

### Effects of L-serine on rotenone-damaged HT22 cell viability

**Fig. 5:**
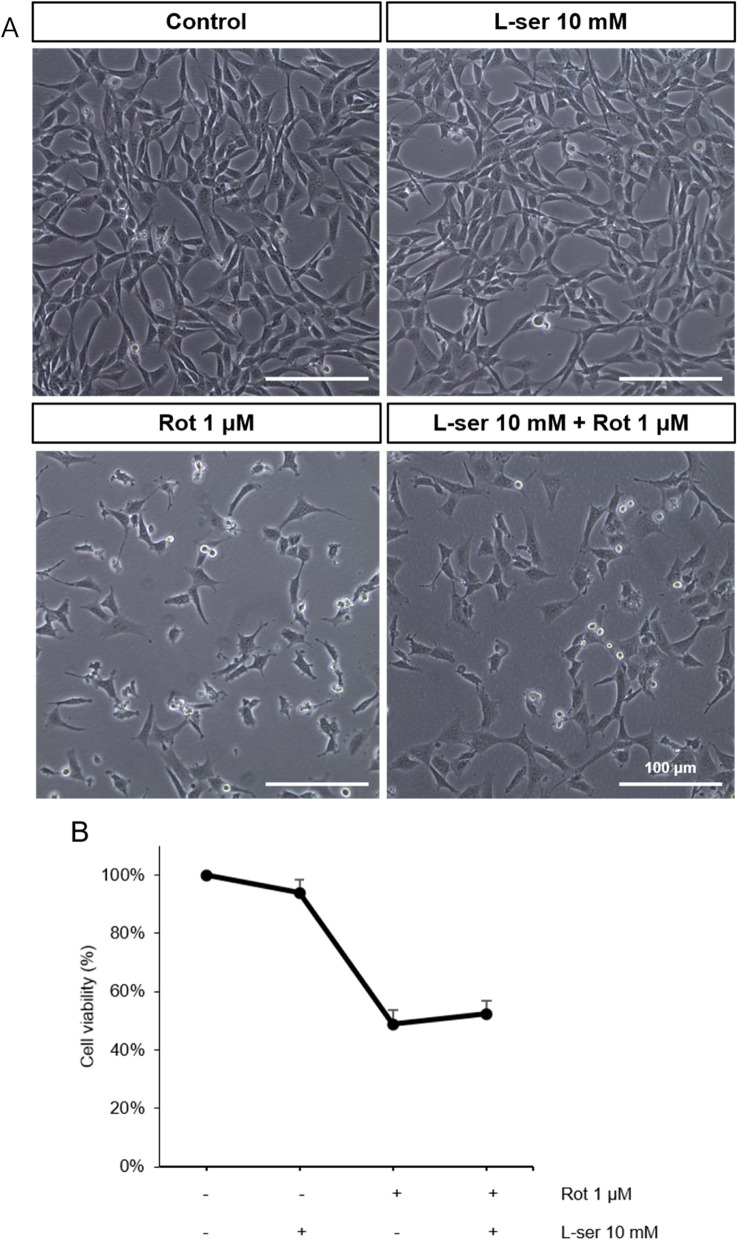
L-serine affects HT22 cell viability, which was negatively affected by rotenone. Untreated HT22 cells, treatment with 10 mM of L-serine for 24 h, treatment with 1 μM of rotenone for 19 h and 5 h treatment with 10 mM L-serine (**A**, **B**). Morphological changes in HT22 cells were observed with an phase-contrast microscope with or without rotenone or L-serine (**A**). Cell viability of HT22 was assessed by CCK-8 assay (**B**). The reduced cell viability was slightly recovered in the L-serine pre-treatment group

Finally, the protective effect of L-serine on apoptosis caused by oxidative stress in HT22 cells was investigated. CCK-8 assay was used to determine cell viability. Cell viability was reduced by half in the rotenone-treated group compared to the untreated control group. In the L-serine pretreatment group, it increased by about 0.1 times compared to the rotenone alone treatment group. The cell viability showed a slight increase in the protective effect of L-serine (Fig. 5A, B).

## Discussion

Mitochondrial activity is one of the key metabolic pathways required for neuronal survival. Oxidative stress reduces the function of mitochondria and the mitochondrial dysfunction is closely related to other cellular organelles’ function. As well known, Acute mitochondrial dysfunction activates autophagy and lysosomal biosynthesis in cells. This is caused by the stimulating autophagosome, triggering a program that removes defective mitochondria. However, if this program continues, the cells eventually run out of mitochondria, which threatens their survival. Thus, mitochondrial dysfunction eventually causes the cell to inhibit the lysosomal functions (Deus, Yambire et al. 2020). Lysosomes are large acidic organelles that degrades abnormal organelles including mitochondria, lipids, aggregated proteins, mainly through autophagy. Autophagy plays a key role in the degradation of misfolded or damaged proteins that are associated with neurodegeneration (Dunlop, 2020). In the autophagic process, cytoplasmic materials engulf into autophagosomes and are degraded by lysosomal enzymes at the end of fusion of lysosomes with autophagosomes (Kuma et al., 2004). Rotenone-induced mitochondrial dysfunction and cell damage have been reported in many papers (Higuchi et al., 1998, Barrientos and Moraes 1999, Isenberg 2000, Chauvin et al., 2001, Sherer et al., 2002). L-serine regulates neurotoxicity by activating the glycine receptor, which is a significant ion channel receptor in the brain. Additionally, researchers showed a significant protective effect of L-serine on the nervous system (De Koning et al., 2003, Sun et al., 2014). In our results, rotenone-induced cellular and mitochondrial ROS was attenuated by pretreatment with 10 mM of L-serine (Figs. 1 and 2). L-serine showed antioxidative and protective effects on mitochondria in HT22 cells damaged by rotenone as supporting previous reports.

Substrates that are no longer required by the cells should be degraded through the autophagy process. However, in the group treated with rotenone alone, substrates accumulated without being degraded, which was protected or restored in the L-serine pretreatment group based on electron microscope images (Fig. 3). This showed the same tendency in the lysosomal activity assay (Fig. 4). It suggests that L-serine possesses antioxidative and protective effects on lysosomes in HT22 cells damaged by rotenone. Cell viability also showed a slight increase suggesting protective effect of L-serine (Fig. 5).

## Conclusion

In conclusion, the results in this study showed that the oxidative stress caused by rotenone in HT22 cells was protected by L-serine. L-serine reduces ROS in cells and specially in the damaged mitochondria. Lysosomal functions and structures were also restored with L-serine. Therefore, the results of this study suggest evidence that L-serine could be used for neuronal protection by the reduction of ROS.

## Data Availability

The datasets used and/or analyzed during the current study are available from the corresponding author on reasonable request.
